# Advances of Molecular Imaging in Epilepsy

**DOI:** 10.1007/s11910-016-0660-7

**Published:** 2016-04-25

**Authors:** Marian Galovic, Matthias Koepp

**Affiliations:** Department of Clinical and Experimental Epilepsy, UCL Institute of Neurology, 33 Queen Square, London, WC1N 3BG United Kingdom; Epilepsy Society MRI Unit, Epilepsy Society, Chalfont St Peter, Buckinghamshire, United Kingdom

**Keywords:** Positron-emission tomography, Epilepsy, Seizures, Neuroimaging

## Abstract

Positron emission tomography (PET) is a neuroimaging method that offers insights into the molecular functioning of a human brain. It has been widely used to study metabolic and neurotransmitter abnormalities in people with epilepsy. This article reviews the development of several PET radioligands and their application in studying the molecular mechanisms of epilepsy. Over the last decade, tracers binding to serotonin and γ-aminobutyric acid (GABA) receptors have been used to delineate the location of the epileptic focus. PET studies have examined the role of opioids, cannabinoids, acetylcholine, and dopamine in modulating neuronal hyperexcitability and seizure termination. In vivo analyses of drug transporters, e.g., P-glycoprotein, have increased our understanding of pharmacoresistance that could inform new therapeutic strategies. Finally, PET experiments targeting neuroinflammation and glutamate receptors might guide the development of novel biomarkers of epileptogenesis.

## Introduction

Positron emission tomography (PET) is one of the first neuroimaging techniques that offered unprecedented insights into the molecular functioning of a living human brain. It involves the injection of a positron-emitting radioligand (tracer) and the detection of coincident gamma waves within a scanner. The resulting image represents the spatial distribution of the tracer within the brain.

The 1980s saw the first major implementation of PET in epilepsy after the development of [^18^F]fluorodeoxyglucose (FDG) to measure local brain glucose metabolism [1]. It was soon recognized that focal interictal hypometabolism correlated with the localization of the epileptic focus, and the method was widely implemented in presurgical epilepsy evaluation [2]. Although FDG remains the workhorse radioligand used in clinical PET imaging, the relevance of FDG PET has slowly diminished after the advent of high-resolution magnetic resonance tomography (MRI).

The last decade brought a gradual shift to the development of receptor-specific ligands that would reveal the in vivo neurochemistry of the epileptic brain. Only few tracers have demonstrated acceptable brain penetration, low-nonspecific binding, high affinity, and target selectivity to progress into clinical studies (Table 1). This review highlights the latest major trends, focusing on PET ligands, which have been used in vivo in people with epilepsy.

**Table 1:** Overview of radioligands recently used in epilepsy

Group	Target	Radioligand
GABA	GABA_A_ receptor	[^11^C]flumazenil-PET
		[^18^F]flumazenil-PET
Glutamate	NMDA receptor	[^18^F]GE-179
Drug transporters	P-glycoprotein	[^11^C]verapamil
Inflammation	TSPO	[^11^C]PBR28
		[^18^F]PBR111
		[^11^C]*(R)*-PK11195
Serotonin, inflammation	Tryptophan metabolism	α-[^11^C]methyl-l-tryptophan
Serotonin	5-HT_1A_ receptor	[^18^F]MPPF
		[^11^C]WAY-100635
		[^18^F]FCWAY
	Serotonin transporter	[^11^C]DASB
Dopamine	Presynaptic dopamine	[^18^F]fluoro-l-DOPA
	D_2_/D_3_ receptor	[^18^F]fallypride
	D_1_ receptor	[^11^C]SCH23390
	Dopamine transporter	[^11^C]PE2I
Cannabinoids	CB_1_ receptor	[^18^F]MK-9470
		[^11^C]MePPEP
Opioids	μ, δ and κ opioid receptors	[^11^C]diprenorphine
	μ opioid receptors	[^11^C]carfentanil
	δ opioid receptors	[^11^C]methylnaltrindole
Acetylcholine	Nicotinic ACh receptor	[^18^F]fluoro-A-85380

## γ-Aminobutyric Acid

Since the late 1990s, a large number of PET studies have described the use of [^11^C]flumazenil PET in epilepsy. This radioligand binds to the benzodiazepine site on the γ-aminobutyric acid (GABA_A_) receptor complex and has shown promising results in the localization and lateralization of the epileptic focus [3]. Its binding is reduced in hippocampal sclerosis and vascular lesios but can be elevated in areas of dysgenesis.

An interesting finding has recently been obtained with [^11^C]flumazenil PET in a mixed group of nonlesional focal epilepsy patients. Increased seizure frequency was inversely correlated with uptake in the frontal piriform cortex [4]. This result is particularly intriguing, because it was independent of the site of seizure onset and has been reproduced with EEG-fMRI and morphometric MRI [4, 5]. Similarly, a crucial epileptogenic area has been described in the prepiriform cortex of rats and monkey and termed “area tempestas” [6]. Nevertheless, the tracer has not seen wide application in clinical routine, mainly due to its short half-life. The recent development of an [^18^F] alternative might overcome these restrictions [7].

## Glutamate

Glutamate is the main excitatory neurotransmitter of the central nervous system and is released shortly before and during epileptic seizures [8].

The N-methyl-d-asparate (NMDA) glutamate receptor is not only involved in long-term potentiation and learning but has also been implicated in excitotoxic neuronal damage and epileptogenesis [9, 10]. Multiple attempts of developing suitable PET ligands for imaging NMDA receptors in epilepsy failed [6]. Experiments in temporal lobe epilepsy (TLE) with these tracers, e.g., with [^11^C]-labeled ketamine, usually showed reduced tracer uptake, possibly reflecting either reduced NMDA receptor density, reduced perfusion, or focal atrophy [11].

### [^18^F]GE-179 PET

**Fig. 1:**
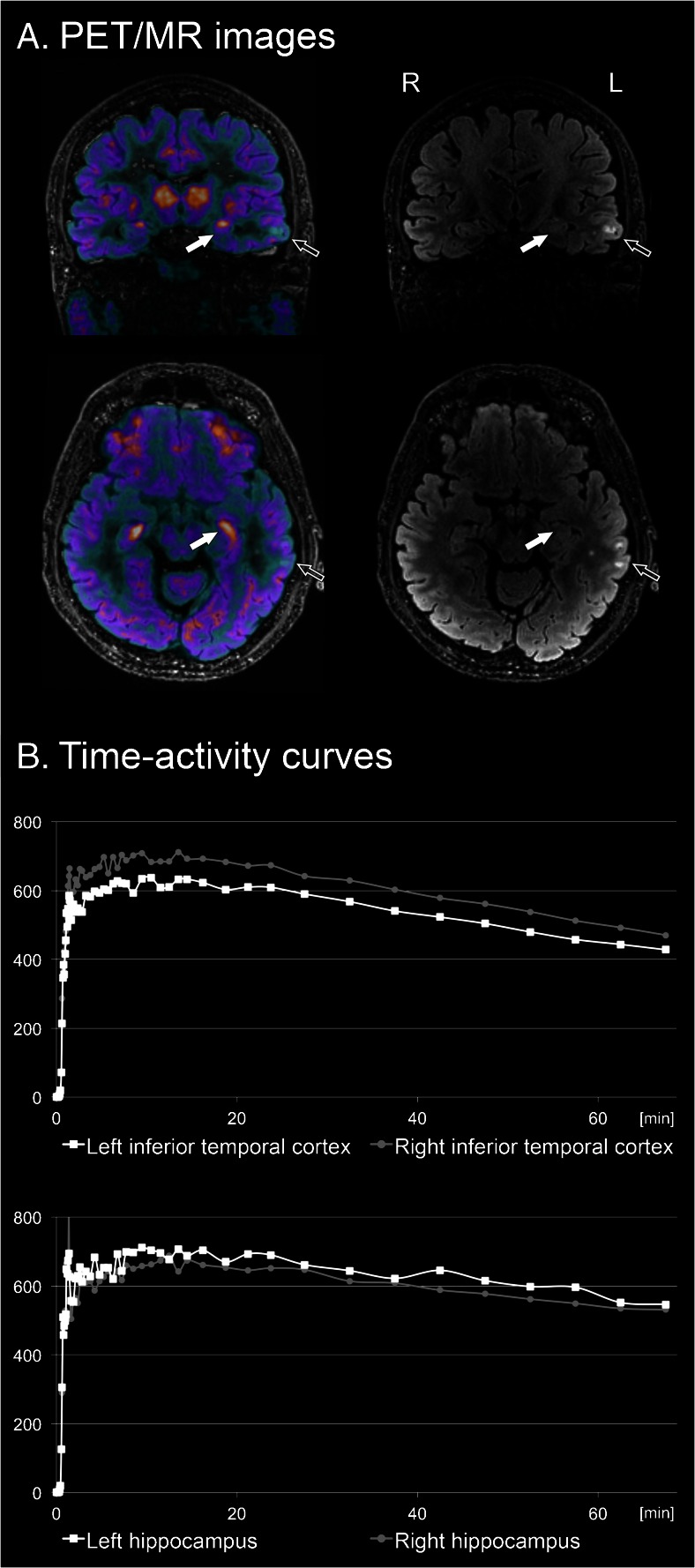
[^18^F]GE-179 PET/MR scan in a 57-year old temporal lobe epilepsy patient with signal abnormality in left inferior temporal cortex. **a** PET/MR fusion image (*left*) and MR FLAIR sequence (*right*) displaying parts of the lesion (*empty arrow*) and the hippocampus (*full arrow*). **b** Bilateral comparison of time-activity curves (*TACs*) in the inferior temporal cortex (*above*) and the hippocampus (*below*). Both visual analysis and TACs show reduced tracer uptake in the lesioned temporal cortex and slightly increased uptake in the ipsilateral hippocampus. Hypothetically, the extralesional increase of NMDA-receptor activation in the ipsilateral hippocampus could point to ongoing epileptogenesis and prospective studies will be needed to prove this assumption

We developed [^18^F]GE-179, which binds to the phencyclidine site within the NMDA ion channel pore, thus indicating the activated state of the receptor [12]. In a pilot study, eight epilepsy patients not taking antidepressants had a significantly increased global radioligand binding and four had focally increased signal compared to controls suggesting increased NMDA receptor activation (Fig. 1). This could point to ongoing epileptogenesis in the group of refractory epilepsy patients [13••]. The most surprising finding was a markedly decreased global binding in three epilepsy patients taking antidepressants, which fell below that of healthy controls. The potential influence of antidepressants and, possibly, depression on activation of NMDA receptors warrants further research.

## Multidrug transporters

Around one third of epilepsy patients are resistant to treatment with antiepileptic drugs (AEDs). In these patients, pharmacoresistance is not limited to a single drug but affects drugs acting through diverse mechanisms. Multidrug efflux transporters such as P-glycoprotein might limit intracellular AED concentration by pumping them from the cell and thus contribute to drug resistance [14]. Pharmacoresistant epilepsy was associated with a genotype leading to increased expression of P-glycoprotein [15], and increased P-glycoprotein expression has been observed in hippocampal tissue of refractory epilepsy patients and in rat epilepsy models [16, 17].

### [^11^C]verapamil PET

To study the contribution of P-glycoprotein to pharmacoresistance in vivo, an experimental PET protocol involving a radiolabeled P-glycoprotein substrate, [^11^C]verapamil, and partial, half-maximum P-glycoprotein blockage with tariquidar has been proposed [14]. The brain uptake of [^11^C]verapamil would correlate with the magnitude of P-glycoprotein action at the blood–brain barrier. Studies in healthy controls confirmed a uniform distribution of P-glycoprotein activity throughout the brain without regional differences [18, 19]. A pilot project in seven epilepsy patients found a trend for lower [^11^C]verapamil influx constants, indicating increased P-glycoprotein function, in the temporal cortex ipsilateral to the epileptic focus [20]. Although these results were obtained in a small sample and failed to reach significance, they informed further evaluation of the tracer in 14 drug refractory epilepsy patients, 8 seizure-free patients, and 13 healthy controls [21••]. Pharmacoresistant patients had a lower baseline influx rate constant (K_1_) corresponding to a higher P-glycoprotein activity. These results were significant in the bilateral hippocampus, ipsilateral amygdala, fusiform gyrus, inferior temporal gyrus, and middle temporal gyrus. Seizure frequency was positively correlated with P-glycoprotein activity in the hippocampus and on a whole brain level. The findings of [^11^C]verapamil PET were validated in ex vivo specimen of five patients who underwent epilepsy surgery. Equivalent results were externally reproduced in a subsequent study of 11 epilepsy patients [22].

A follow-up [^11^C]verapamil project analyzed P-glycoprotein activity in seven patients undergoing epilepsy surgery [23]. Increased P-glycoprotein function before surgery and a decrease postoperatively were associated with optimal surgical outcome. The results also indicated that P-glycoprotein expression responded dynamically to therapeutic procedures or changes in seizure frequency.

These studies provide intriguing evidence for an involvement of multidrug efflux transporters in pharmacoresistant epilepsy. If confirmed, [^11^C]verapamil PET could be used to identify patients with P-glycoprotein overactivity who might benefit from novel treatment strategies aimed at inhibiting or modulating P-glycoprotein activity [21••]. The main limitation is, however, that the authors could not distinguish whether P-glycoprotein overactivity was a cause or consequence of increased seizure frequency [24]. Another difficulty arose from high uptake in the choroid plexus, which complicates quantification of PET tracer uptake in the hippocampus. This particular effect was used to outline the choroid plexus using a different P-glycoprotein substrate tracer, [^11^C]-N-desmethyl-loperamide, in a PET study assessing translocator protein (TSPO) binding in the hippocampus [25].

## Inflammation

Inflammatory mediators were found in human and rodent epileptic tissue and it was demonstrated that experimental seizures caused a rapid and pronounced inflammatory reaction [26–28]. Additionally, some antiepileptic drugs were shown to have antiinflammatory properties [29].

### Translocator protein positron emission tomography

Several PET tracers have been developed to target neuroinflammation [30]. The most commonly used radioligands are [^11^C]PK11195 and [^11^C]PBR28 that bind to TSPO, a marker of activated microglia [26]. The concentration of TSPO is very low in healthy brain tissue; however, a marked increase can be observed in experimental models inducing neuroinflammation in the form of microglial activation [31, 32]. This increase can be reliably measured with TSPO PET tracers [31, 32]. In rat models of epileptogenesis, increased TSPO expression pointing to neuroinflammation can be demonstrated with PET [28]. The inflammatory process peaks 2 weeks after initial status epilepticus but limbic activation of microglia persists into the chronic phase [26].

The first human investigations with TSPO PET were performed in single subjects or small case series with presumed inflammatory epileptic encephalopathies. In an elegant study, unilaterally increased TSPO expression has been demonstrated with 11C-PK11195 PET in two patients with histologically confirmed Rasmussen’s encephalitis, substantiating the role of neuroinflammation in this syndrome [33]. Similarly, increased tracer binding has been demonstrated in a case with seizures due to cerebral vasculitis and in another patient with an epileptic encephalitis of unknown etiology [34, 35].

Two recent well-powered studies in temporal lobe epilepsy showed increased TSPO radioligand uptake, pointing to activation of microglia, in temporal regions ipsilateral to the epileptic focus and, to a lesser extent, in the ipsilateral thalamus and contralateral temporal lobe [36••, 37]. However, concordance of increased TSPO PET signal with the ictal-EEG onset zone has not been evaluated and the role of this technique as a diagnostic localizing tool is uncertain. Nevertheless, these in vivo findings give support to a local neuroinflammation in human epileptic cortex. Although the causal role of brain inflammation in generating seizures still needs to be confirmed, these observations might provide a rationale for antiinflammatory treatment in some epilepsy patients.

## Serotonin

The influence of serotonin (5-HT) on hyperexcitability has been studied for decades. Several animal models demonstrated that elevating extracellular serotonin levels inhibits seizures, mainly acting through the 5-HT_1A_ receptor subtype [38]. Conversely, increased levels of serotonin metabolites were found in resected epileptic tissue [39]. The interest in serotonin has led to the development of several suitable PET tracers that study three different aspects of cerebral serotonin function.

### α-[^11^C]methyl-l-tryptophan positron emission tomography

Originally, increased uptake of α-[^11^C]methyl-l-tryptophan ([^11^C]AMT) was thought to reflect increased serotonin synthesis. The downside of this approach is that serotonin metabolism might be disturbed in disease and the findings are difficult to interpret in a pathophysiological context [40]. Additionally, recent evidence in patients with tuberous sclerosis complex (TSC) points to an increased tryptophan metabolism via the kynurenine pathway in the presence of neuroinflammation, leading to the production of proconvulsants [40]. Hence, [^11^C]AMT PET might also reflect the degree of inflammation in neuronal tissue.

The differentiation of epileptogenic and non-epileptogenic tubers was extensively examined with [^11^C]AMT PET and increased tracer binding was consistently demonstrated in tubers that colocalized with ictal EEG findings [41–43]. A recently published large series of 191 TSC patients demonstrated excellent agreement of [^11^C]AMT PET with ictal EEG findings [44••]. Moreover, [^11^C]AMT PET supplied localizing information in more than half of patients with inconclusive EEG. The results were independent of underlying TSC mutation. A smaller series of 12 TSC patients demonstrated low sensitivity (12 %) but high specificity (100 %) of [^11^C]AMT PET in the prediction of epileptogenic tubers [45•]. Although this series is relatively small, it is one of few [^11^C]AMT PET studies performed outside of Detroit, providing valuable data for external validation of this tracer.

[^11^C]AMT PET has also been applied to intractable childhood epilepsy of other causes [46, 47]. Again, sensitivity for correct localizing information was low, but nearly perfect specificity surpassed that of FDG PET. However, the limiting factor for the clinical use of this promising tracer is the difficult synthesis and short half-life (20 min). Production is currently restricted to a few dedicated centers worldwide, which severely impacts the application of these findings in clinical practice.

### 5-HT_1A_ Receptor Ligands and Tracer for Serotonin Transporter

The density of 5-HT_1A_ receptors has been measured with several PET tracers that differ in their pharmacological properties (Table 1). [^18^F]MPPF is a selective antagonist of 5-HT_1A_ with an affinity close to that of serotonin and is, hence, sensitive to endogenous serotonin variations. Conversely, [^11^C]WAY-100635 and [^18^F]FCWAY are high-affinity agonists of 5-HT_1A_ and they do not compete with endogenous serotonin [48]. Finally, [^11^C]DASB can measure serotonin transporter 5-HTT availability, the main terminator of synaptic serotonin effect [49•].

Decrease of 5-HT_1A_ receptor density ipsilateral to seizure focus is a consistent finding in TLE. Many PET studies using [^18^F]MPPF, [^11^C]WAY-100635, and [^18^F]FCWAY have demonstrated similar results despite using different methodical approaches [50–56]. The decreases were more pronounced in the hippocampus and in areas involved in seizure generation [52, 55]. These findings fit well with the concept of a proconvulsive effect of serotonin depletion.

The use of 5-HT_1A_ receptor PET in presurgical epilepsy evaluation has only been tested in small patient samples. 5-HT_1A_ receptor PET showed decreased temporal binding in more than 80 % of these cases and all pathological decreases were congruent with the lateralization of the ictal onset on EEG [54, 57]. All patients with lateralizing [^18^F]MPPF PET became seizure-free after surgery [57]. These results suggest a higher specificity than FDG PET; however, they remain to be reproduced in larger patient samples. The sensitivity of 5-HT_1A_ receptor PET can be further improved by comparing tracer binding between both cerebral hemispheres using asymmetry indices. This increases the sensitivity to above 90 % with a specificity of 88 % [58].

Some studies have reported HT_1A_ abnormalities beyond the temporal lobe, describing decreased [^18^F]FCWAY binding in the insular cortex and anterior cingulate [51, 52]. Such changes in the limbic areas were significantly more common in epilepsy patients with concomitant depression compared to those without mood disturbances[51, 53]. Also, the magnitude of hippocampal binding inversely correlated with depressive symptoms [59]. These results suggest a common pathomechanism of epilepsy and comorbid depression due to a decrease of serotonin receptors that extends beyond the temporal lobe and affects limbic structures.

A seemingly contradictory finding was demonstrated using [^18^F]MPPF PET, showing increased uptake in insula and raphe nuclei of depressed epilepsy patients [60]. However, this increased binding most likely reflects a decreased extracellular serotonin concentration in epilepsy patients with depression resulting in increased 5-HTT availability.

A recent study examined serotonin transporter 5-HTT function using [^11^C]DASB PET in 13 TLE patients and 16 controls [49•]. There were no regional differences in 5-HTT function between patients and controls. However, epilepsy patients with history of depression had a relatively reduced 5-HTT activity in the ipsilateral insula compared to those without depression. A reduced transporter activity would decrease serotonin reuptake and might represent a compensation mechanism to increase extracellular serotonin concentration. Insular 5-HTT activity correlated with 5-HT_1A_ receptor density measured with [^18^F]FCWAY-PET, indicating that a reduction of 5-HT_1A_ receptors may be associated with decreased reuptake of serotonin. However, this study creates more questions than it answers. It remains unclear why epilepsy patients without depression do not show reduced 5-HTT function and why depressed patients develop mood disorders despite these compensatory mechanisms. The results need to be interpreted with caution especially because contradictory findings were reported in patients with major depression [61].

## Dopamine

“The role of dopamine in epilepsy is intriguing, complex, and unresolved” [62]. Initial input came from experimental data suggesting that dopaminergic neurons in the striatum and substantia nigra were involved in seizure termination [63, 64]. These dopamine-rich nigrostriatal areas are thought to modulate thalamocortical projections to regions involved in epilepsy syndromes [62].

Within the past decade, a number of PET studies demonstrated an abnormal subcortical dopaminergic system in epilepsy. Almost unequivocally, they found a bilaterally reduced dopaminergic function within the basal ganglia. The utilization of several radioligands demonstrated different aspects of dopaminergic dysregulation (Table 1): a presynaptic dopaminergic deficit using [^18^F]fluoro-l-DOPA [65–67], decreased D_2_/D_3_-receptor binding using [^18^F]fallypride [68–71], decreased D_1_-receptor binding using [^11^C]SCH23390 [72], and reduced dopamine transporter activity with [^11^C]PE2I [73–75]. These authors investigated a variety of epilepsy syndromes including temporal lobe epilepsy, juvenile myoclonic epilepsy, idiopathic generalized epilepsy, autosomal dominant nocturnal frontal lobe epilepsy (ADNFLE), and ring chromosome 20 syndrome. Most authors found abnormalities in the striatum, whereas changes in the midbrain (i.e., substantia nigra) were reported in juvenile myoclonic epilepsy [73–75]. Although it is difficult to assess dopaminergic binding outside the basal ganglia, two studies described decreased uptake of the epileptogenic zone of patients with temporal lobe epilepsy [68, 71].

The diversity of methodical approaches and patient populations underlines the robustness of these results. However, the detection of dopaminergic abnormalities independent of the underlying type or pathology of epilepsy suggests that these alterations are a nonspecific reaction to seizures [76].

Taken together, an altered dopaminergic system is commonly seen in epilepsy and might act to impair termination of seizures. Also, the findings highlight the importance of subcortical structures in epilepsy. However, the basal ganglia likely represent only the tip of the iceberg within a more widespread dopamine dysregulation. Nevertheless, it is difficult to ascertain significant extrastriatal changes with PET because cortical binding of dopamine is generally low [62, 76].

## Cannabinoids

Most PET ligand development in this group has focused on cannabinoid receptor 1 (CB_1_), the major cannabinoid receptor in the central nervous system. Several lines of evidence indicate that CB_1_ has anticonvulsant properties that mainly rely on the induction and modulation of protective neuronal mechanisms [77, 78]. A downregulation of CB_1_ during epileptogenesis might impair these mechanisms and facilitate seizures [79, 80]. However, experimental research is not entirely conclusive as others have reported that activation of CB_1_ receptors might be proepileptogenic as CB_1_ antagonists prevented the development of epilepsy [81].

Goffin et al. recently examined the activation of the endocannabinoid system in temporal lobe epilepsy with the CB_1_ radioligand [^18^F]MK-9470 [82]. They found an increased uptake in the temporal lobe ipsilateral to the epileptic cortex and a bilateral decrease in insular CB_1_ binding. However, the authors could not differentiate whether increased CB_1_ availability in the epileptic cortex was an abnormal proconvulsive or a protective anticonvulsive phenomenon.

Quantification of CB_1_ receptor availability using a different radioligand, [^11^C]MePPEP, recently showed good to excellent reproducibility [83] with investigation of CB_1_ receptor-mediated neurotransmission in epilepsy currently under way.

## Opioids

Increased release of endogenous opioids has previously been demonstrated during provoked absences and reading-induced seizures [84, 85]. These findings have underlined the potential role of opioids in the termination of epileptic seizures.

### [^11^C]diprenorphine

A recent study investigated opioid receptor binding using [^11^C]diprenorphine PET after spontaneous seizures [86]. Hammers et al. scanned nine refractory temporal lobe epilepsy patients within hours after spontaneous seizures and rescanned them during the later interictal phase. After a median postictal interval of 8 h, increased binding was found in the ipsilateral temporal pole, fusiform gyrus, and, after correction for the partial volume effect, also in the parahippocampal gyrus [87]. There was a negative correlation of ligand uptake with the postictal interval, pointing to an early increase in binding with a gradual return to normal.

These findings might either indicate an increased expression of opioid receptors, a reduced endogenous opioid tone or both. In context with previous research, the authors favored the first explanation. The most likely sequence after spontaneous seizures starts with a release of endogenous opioids leading to decreased [^11^C]diprenorphine binding [84, 85]. This is followed by a rapid recovery of available receptors and an overshoot in receptor expression, depicted as increased binding after 8 h on PET [86, 87]. Finally, there is a gradual return to normal or low-normal levels during the interictal phase.

## Acetylcholine

ADNFLE is an uncommon familial epilepsy syndrome associated with hypermotor seizures occurring during sleep. Two causative genes have been described, most likely leading to a gain of function of the α4β2 subtype of the nicotinic acetylcholine receptor (nAChR) [88, 89].

Picard et al. examined eight ADNFLE patients using PET with [^18^F]fluoro-A-85380, a high affinity agonist at the α4β2 nAChR [90]. Increased tracer uptake was demonstrated in the epithalamus, ventral mesencephalon, and cerebellum, whereas decreased binding was found in the right dorsolateral prefrontal region. The mesencephalic and thalamic increases point toward an overactivated cholinergic pathway ascending from the brainstem. These findings are particularly interesting in the context of a sleep disorder and might indicate a unique pathogenesis of nocturnal seizures. Conversely, decreased receptor density in the prefrontal region could be due to neuronal loss as part of the frontal lobe epilepsy.

However, some questions remain unanswered. It remains unknown whether the observed changes are a hallmark of ADNFLE or a non-specific consequence of seizures. Moreover, although the authors demonstrated spatial changes in receptor density, the functional consequences of the mutation on nAChR activity remain unclear.

## Conclusions

PET studies have successfully demonstrated a number of molecular functional abnormalities in epilepsy (Table 2). These insights have not only increased our understanding of the underlying mechanisms of seizures but will also improve the diagnostic evaluation of MRI negative patients. Increasing efforts are being put into translating the novel results into the development of new therapeutics. These will specifically target the neurotransmitters and molecular mechanisms demonstrated in PET studies.

**Table 2:** Main findings of recent PET studies in epilepsy

Group	Target	Main findings in epilepsy patients	Author’s interpretation
GABA	GABA_A_ receptor	Inverse correlation of seizure frequency with uptake in the frontal piriform cortex in patients with different sites of seizure onset [4].	The prepiriform cortex might represent a common epileptogenic area independent of the localization of seizure onset.
Glutamate	NMDA receptor	Increased global uptake in patients not on antidepressants. [13••]	Global increase of NMDA receptor activation might reflect ongoing epileptogenesis.
Multidrug transporters	P-glycoprotein	Increased P-glycoprotein activity in pharmacoresistant patients, particularly in mesiotemporal areas [21••, 22]	Increased P-glycoprotein activity could contribute to multidrug resistance by reducing the intracellular concentration of antiepileptic drugs.
Inflammation	TSPO	Increased uptake in ipsilateral temporal lobe and, to a lesser extent, in ipsilateral thalamus and contralateral temporal lobe. [36••, 37]	Increased TSPO expression points to activation of microglia and an inflammatory reaction in epilepsy patients that could induce epileptogenesis.
Serotonin, inflammation	Tryptophan metabolism	Increased uptake in epileptic vs. non-epileptic brain tubers in TSC. Increased uptake in the epileptic focus of children with intractable epilepsy. Low sensitivity but high specificity of these findings [44••, 45•, 46, 47]	[^11^C]AMT-PET adds valuable information on the location of the epileptic focus. It might reflect increased tryptophan metabolism that indicates the local production of proconvulsants.
Serotonin	5-HT_1A_ receptor	Reduced uptake ipsilaterally to seizure focus, particularly in the hippocampus. Decreased uptake in insular cortex and anterior cingulate in depressed epilepsy patients [50–59]	Adds lateralizing information with higher specificity than FDG-PET. A widespread reduction of serotonin receptors extending beyond the temporal lobe might indicate a pathomechanism of comorbid depression.
	Serotonin transporter	Reduced uptake in ipsilateral insula in epilepsy patients with depression [49•]	Decreased serotonin reuptake might represent a compensatory mechanism for low serotonin levels in comorbid depression.
Dopamine	Presynaptic dopamine, D_1_/D_2_/D_3_ receptor, dopamine transporter	Bilaterally reduced uptake in basal ganglia, particularly striatum and substantia nigra [65–75]	An altered dopaminergic neurotransmission might impair termination of seizures.
Cannabinoids	CB_1_ receptor	Increased uptake in ipsilateral temporal lobe; decreased uptake in bilateral insula [82]	Supports dysregulation of cannabinoids in epilepsy that could represent a pro or anticonvulsive phenomenon.
Opioids	μ, δ. and κ opioid receptors	Reduced radioligand uptake during absence and reading-induced seizures. Increased uptake 8 h after spontaneous seizures [84–87]	Opioid release during seizures might contribute to seizure termination. This is likely followed by an early interictal overexpression of opioid receptors.
Acetylcholine	Nicotinic ACh receptor	Increased uptake in epithalamus, ventral mesencephalon, and cerebellum in ADNFLE patients. Decreased uptake in prefrontal cortex [90]	Thalamic and mesencephalic findings may indicate a unique mechanism of nocturnal seizures in ADNFLE. Reduced prefrontal receptor density could be due to neuronal loss.

A central challenge will be to tackle the problem of epileptogenesis. The long latent period between an initial insult and the subsequent development of seizures offers plenty of time for diagnostic and therapeutic interventions [91]. PET might play an important role as a biomarker by revealing the molecular processes involved in the development of epileptogenesis.

## References

[1] Phelps ME, Huang SC, Hoffman EJ, Selin C, Sokoloff L, Kuhl DE (1979). Tomographic measurement of local cerebral glucose metabolic rate in humans with (F-18)2-fluoro-2-deoxy-D-glucose: validation of method. Ann Neurol.

[2] Kuhl DE, Engel J, Phelps ME, Selin C (1980). Epileptic patterns of local cerebral metabolism and perfusion in humans determined by emission computed tomography of 18FDG and 13NH3. Ann Neurol.

[3] la Fougère C, Rominger A, Förster S, Geisler J, Bartenstein P (2009). PET and SPECT in epilepsy: a critical review. Epilepsy Behav.

[4] Laufs H, Richardson MP, Salek-Haddadi A, Vollmar C, Duncan JS, Gale K (2011). Converging PET and fMRI evidence for a common area involved in human focal epilepsies. Neurology.

[5] Centeno M, Vollmar C, Stretton J, Symms MR, Thompson PJ, Richardson MP (2014). Structural changes in the temporal lobe and piriform cortex in frontal lobe epilepsy. Epilepsy Res.

[6] Piredda S, Gale K (1985). A crucial epileptogenic site in the deep prepiriform cortex. Nature.

[7] Vivash L, Gregoire M-C, Lau EW, Ware RE, Binns D, Roselt P (2013). 18F-flumazenil: a γ-aminobutyric acid A-specific PET radiotracer for the localization of drug-resistant temporal lobe epilepsy. J Nucl Med.

[8] During MJ, Spencer DD (1993). Extracellular hippocampal glutamate and spontaneous seizure in the conscious human brain. Lancet.

[9] Yeh GC, Bonhaus DW, Nadler JV, McNamara JO (1989). N-methyl-D-aspartate receptor plasticity in kindling: quantitative and qualitative alterations in the N-methyl-D-aspartate receptor-channel complex. Proc Natl Acad Sci U S A.

[10] Stasheff SF, Anderson WW, Clark S, Wilson WA (1989). NMDA antagonists differentiate epileptogenesis from seizure expression in an in vitro model. Science.

[11] Kumlien E, Hartvig P, Valind S, Oye I, Tedroff J, Långström B (1999). NMDA-receptor activity visualized with (S)-[N-methyl-11C]ketamine and positron emission tomography in patients with medial temporal lobe epilepsy. Epilepsia.

[12] McGinnity CJ, Hammers A, Riaño Barros DA, Luthra SK, Jones PA, Trigg W (2014). Initial evaluation of 18F-GE-179, a putative PET Tracer for activated N-methyl D-aspartate receptors. J Nucl Med.

[13.••] McGinnity CJ, Koepp MJ, Hammers A, Riaño Barros DA, Pressler RM, Luthra S (2015). NMDA receptor binding in focal epilepsies. J Neurol Neurosurg Psychiatr.

[14] Feldmann M, Koepp M (2012). P-glycoprotein imaging in temporal lobe epilepsy: in vivo PET experiments with the Pgp substrate [11C]-verapamil. Epilepsia.

[15] Siddiqui A, Kerb R, Weale ME, Brinkmann U, Smith A, Goldstein DB (2003). Association of multidrug resistance in epilepsy with a polymorphism in the drug-transporter gene ABCB1. N Engl J Med.

[16] Volk HA, Burkhardt K, Potschka H, Chen J, Becker A, Loscher W (2004). Neuronal expression of the drug efflux transporter P-glycoprotein in the rat hippocampus after limbic seizures. Neuroscience.

[17] Sisodiya SM, Lin W-R, Harding BN, Squier MV, Thom M (2002). Drug resistance in epilepsy: expression of drug resistance proteins in common causes of refractory epilepsy. Brain.

[18] Eyal S, Ke B, Muzi M, Link JM, Mankoff DA, Collier AC (2010). Regional P-glycoprotein activity and inhibition at the human blood–brain barrier as imaged by positron emission tomography. Clin Pharmacol Ther.

[19] Wagner CC, Bauer M, Karch R, Feurstein T, Kopp S, Chiba P (2009). A pilot study to assess the efficacy of tariquidar to inhibit P-glycoprotein at the human blood–brain barrier with (R)-11C-verapamil and PET. J Nucl Med.

[20] Langer O, Bauer M, Hammers A, Karch R, Pataraia E, Koepp MJ (2007). Pharmacoresistance in epilepsy: a pilot PET study with the P-glycoprotein substrate R-[(11)C]verapamil. Epilepsia.

[21.••] Feldmann M, Asselin M-C, Liu J, Wang S, McMahon A, Anton-Rodriguez J (2013). P-glycoprotein expression and function in patients with temporal lobe epilepsy: a case–control study. Lancet Neurol.

[22] Shin JW, Chu K, Shin SA, Jung KH, Lee ST, Lee YS, et al. Clinical applications of simultaneous PET/MR imaging using (R)-[11C]-verapamil with cyclosporine A: preliminary results on a surrogate marker of drug-resistant epilepsy. AJNR Am J Neuroradiol. 2015. 10.3174/ajnr.A4566 PMC7960172 26585254

[23] Bauer M, Karch R, Zeitlinger M, Liu J, Koepp MJ, Asselin M-C (2014). In vivo P-glycoprotein function before and after epilepsy surgery. Neurology.

[24] Gidal BE (2014). P-glycoprotein expression and pharmacoresistant epilepsy: cause or consequence?. Epilepsy Curr.

[25] Seneca N, Zoghbi SS, Liow J-S, Kreisl W, Herscovitch P, Jenko K, et al. Human brain imaging and radiation dosimetry of 11C-N-desmethyl-loperamide, a PET radiotracer to measure the function of P-glycoprotein. J Nucl Med. 2009;50:807–13. 10.2967/jnumed.108.058453 PMC2792991 19372478

[26] Amhaoul H, Hamaide J, Bertoglio D, Reichel SN, Verhaeghe J, Geerts E (2015). Brain inflammation in a chronic epilepsy model: evolving pattern of the translocator protein during epileptogenesis. Neurobiol Dis.

[27] Das A, Wallace GC, Holmes C, McDowell ML, Smith JA, Marshall JD (2012). Hippocampal tissue of patients with refractory temporal lobe epilepsy is associated with astrocyte activation, inflammation, and altered expression of channels and receptors. Neuroscience.

[28] Dedeurwaerdere S, Callaghan PD, Pham T, Rahardjo GL, Amhaoul H, Berghofer P (2012). PET imaging of brain inflammation during early epileptogenesis in a rat model of temporal lobe epilepsy. EJNMMI Res.

[29] Kim J-E, Choi H-C, Song H-K, Jo S-M, Kim D-S, Choi S-Y (2010). Levetiracetam inhibits interleukin-1 beta inflammatory responses in the hippocampus and piriform cortex of epileptic rats. Neurosci Lett.

[30] Amhaoul H, Staelens S, Dedeurwaerdere S (2014). Imaging brain inflammation in epilepsy. Neuroscience.

[31] Ory D, Planas A, Dresselaers T, Gsell W, Postnov A, Celen S (2015). PET imaging of TSPO in a rat model of local neuroinflammation induced by intracerebral injection of lipopolysaccharide. Nucl Med Biol.

[32] Sandiego CM, Gallezot J-D, Pittman B, Nabulsi N, Lim K, Lin S-F (2015). Imaging robust microglial activation after lipopolysaccharide administration in humans with PET. Proc Natl Acad Sci U S A.

[33] Banati RB, Goerres GW, Myers R, Gunn RN, Turkheimer FE, Kreutzberg GW (1999). [11C](R)-PK11195 positron emission tomography imaging of activated microglia in vivo in Rasmussen's encephalitis. Neurology.

[34] Kumar A, Chugani HT, Luat A, Asano E, Sood S (2008). Epilepsy surgery in a case of encephalitis: use of 11C-PK11195 positron emission tomography. Pediatr Neurol.

[35] Goerres GW, Revesz T, Duncan J, Banati RB (2001). Imaging cerebral vasculitis in refractory epilepsy using [(11)C](R)-PK11195 positron emission tomography. AJR Am J Roentgenol.

[36.••] Gershen LD, Zanotti-Fregonara P, Dustin IH, Liow JS, Hirvonen J, Kreisl WC (2015). Neuroinflammation in temporal lobe epilepsy measured using positron emission tomographic imaging of translocator protein. JAMA Neurol.

[37] Hirvonen J, Kreisl WC, Fujita M, Dustin I, Khan O, Appel S (2012). Increased in vivo expression of an inflammatory marker in temporal lobe epilepsy. J Nucl Med.

[38] Bagdy G, Kecskemeti V, Riba P, Jakus R (2007). Serotonin and epilepsy. J Neurochem.

[39] Louw D, Sutherland GR, Glavin GB, Girvin J (1989). A study of monoamine metabolism in human epilepsy. Can J Neurol Sci.

[40] Chugani DC (2011). α-methyl-L-tryptophan: mechanisms for tracer localization of epileptogenic brain regions. Biomarkers Med.

[41] Chugani DC, Chugani HT, Muzik O, Shah JR, Shah AK, Canady A (1998). Imaging epileptogenic tubers in children with tuberous sclerosis complex using alpha-[11C]methyl-L-tryptophan positron emission tomography. Ann Neurol.

[42] Asano E, Chugani DC, Muzik O, Shen C, Juhász C, Janisse J (2000). Multimodality imaging for improved detection of epileptogenic foci in tuberous sclerosis complex. Neurology.

[43] Kagawa K, Chugani DC, Asano E, Juhász C, Muzik O, Shah A (2005). Epilepsy surgery outcome in children with tuberous sclerosis complex evaluated with alpha-[11C]methyl-L-tryptophan positron emission tomography (PET). J Child Neurol.

[44.••] Chugani HT, Luat AF, Kumar A, Govindan R, Pawlik K, Asano E (2013). α-[11C]-methyl-L-tryptophan—PET in 191 patients with tuberous sclerosis complex. Neurology.

[45.•] Rubí S, Costes N, Heckemann RA, Bouvard S, Hammers A, Martí Fuster B (2013). Positron emission tomography with α-[11C]methyl-L-tryptophan in tuberous sclerosis complex-related epilepsy. Epilepsia.

[46] Juhász C, Chugani DC, Padhye UN, Muzik O, Shah A, Asano E (2004). Evaluation with alpha-[11C]methyl-L-tryptophan positron emission tomography for reoperation after failed epilepsy surgery. Epilepsia.

[47] Juhász C, Chugani DC, Muzik O, Shah A, Asano E, Mangner TJ (2003). Alpha-methyl-L-tryptophan PET detects epileptogenic cortex in children with intractable epilepsy. Neurology.

[48] Garibotto V, Picard F (2013). Nuclear medicine imaging in epilepsy. Epileptologie.

[49.•] Martinez A, Finegersh A, Cannon DM, Dustin I, Nugent A, Herscovitch P (2013). The 5-HT1A receptor and 5-HT transporter in temporal lobe epilepsy. Neurology.

[50] Toczek MT, Carson RE, Lang L, Ma Y, Spanaki MV, Der MG (2003). PET imaging of 5-HT1A receptor binding in patients with temporal lobe epilepsy. Neurology.

[51] Savic I, Lindstrom P, Gulyás B, Halldin C, Andrée B, Farde L (2004). Limbic reductions of 5-HT1A receptor binding in human temporal lobe epilepsy. Neurology.

[52] Giovacchini G, Toczek MT, Bonwetsch R, Bagic A, Lang L, Fraser C (2005). 5-HT 1A receptors are reduced in temporal lobe epilepsy after partial-volume correction. J Nucl Med.

[53] Hasler G, Bonwetsch R, Giovacchini G, Toczek MT, Bagic A, Luckenbaugh DA (2007). 5-HT1A receptor binding in temporal lobe epilepsy patients with and without major depression. BPS.

[54] Liew CJ, Lim Y-M, Bonwetsch R, Shamim S, Sato S, Reeves-Tyer P (2009). 18F-FCWAY and 18F-FDG PET in MRI-negative temporal lobe epilepsy. Epilepsia.

[55] Merlet I, Ostrowsky K, Costes N, Ryvlin P, Isnard J, Faillenot I (2004). 5-HT1A receptor binding and intracerebral activity in temporal lobe epilepsy: an [18F]MPPF-PET study. Brain.

[56] Merlet I, Ryvlin P, Costes N, Dufournel D, Isnard J, Faillenot I (2004). Statistical parametric mapping of 5-HT1A receptor binding in temporal lobe epilepsy with hippocampal ictal onset on intracranial EEG. Neuroimage.

[57] Didelot A, Ryvlin P, Lothe A, Merlet I, Hammers A, Mauguière F (2008). PET imaging of brain 5-HT1A receptors in the preoperative evaluation of temporal lobe epilepsy. Brain.

[58] Didelot A, Mauguière F, Redouté J, Bouvard S, Lothe A, Reilhac A (2010). Voxel-based analysis of asymmetry index maps increases the specificity of 18F-MPPF PET abnormalities for localizing the epileptogenic zone in temporal lobe epilepsies. J Nucl Med.

[59] Theodore WH, Hasler G, Giovacchini G, Kelley K, Reeves-Tyer P, Herscovitch P (2007). Reduced hippocampal 5HT1A PET receptor binding and depression in temporal lobe epilepsy. Epilepsia.

[60] Lothe A, Didelot A, Hammers A, Costes N, Saoud M, Gilliam F (2008). Comorbidity between temporal lobe epilepsy and depression: a [18F]MPPF PET study. Brain.

[61] Cannon DM, Ichise M, Rollis D, Klaver JM, Gandhi SK, Charney DS (2007). Elevated serotonin transporter binding in major depressive disorder assessed using positron emission tomography and [11C]DASB; comparison with bipolar disorder. BPS.

[62] Haut SR, Albin RL (2008). Dopamine and epilepsy: hints of complex subcortical roles. Neurology.

[63] Velísková J, Moshé SL (2006). Update on the role of substantia nigra pars reticulata in the regulation of seizures. Epilepsy Curr.

[64] Deransart C, Riban V, Lê B, Marescaux C, Depaulis A (2000). Dopamine in the striatum modulates seizures in a genetic model of absence epilepsy in the rat. Neuroscience.

[65] Biraben A, Semah F, Ribeiro M-J, Douaud G, Remy P, Depaulis A (2004). PET evidence for a role of the basal ganglia in patients with ring chromosome 20 epilepsy. Neurology.

[66] Bouilleret V, Semah F, Biraben A, Taussig D, Chassoux F, Syrota A (2005). Involvement of the basal ganglia in refractory epilepsy: an 18F-fluoro-L-DOPA PET study using 2 methods of analysis. J Nucl Med.

[67] Bouilleret V, Semah F, Chassoux F, Mantzaridez M, Biraben A, Trebossen R (2008). Basal ganglia involvement in temporal lobe epilepsy: a functional and morphologic study. Neurology.

[68] Werhahn KJ, Landvogt C, Klimpe S, Buchholz H-G, Yakushev I, Siessmeier T (2006). Decreased dopamine D2/D3-receptor binding in temporal lobe epilepsy: an [18F]fallypride PET study. Epilepsia.

[69] Yakushev IY, Dupont E, Buchholz H-G, Tillmanns J, Debus F, Cumming P (2010). In vivo imaging of dopamine receptors in a model of temporal lobe epilepsy. Epilepsia.

[70] Landvogt C, Buchholz H-G, Bernedo V, Schreckenberger M, Werhahn KJ (2010). Alteration of dopamine D2/D3 receptor binding in patients with juvenile myoclonic epilepsy. Epilepsia.

[71] Bernedo Paredes VE, Buchholz H-G, Gartenschläger M, Breimhorst M, Schreckenberger M, Werhahn KJ (2015). Reduced D2/D3 receptor binding of extrastriatal and striatal regions in temporal lobe epilepsy. PLoS ONE.

[72] Fedi M, Berkovic SF, Scheffer IE, O'Keefe G, Marini C, Mulligan R (2008). Reduced striatal D1 receptor binding in autosomal dominant nocturnal frontal lobe epilepsy. Neurology.

[73] Ciumas C, Wahlin T-BR, Jucaite A, Lindstrom P, Halldin C, Savic I (2008). Reduced dopamine transporter binding in patients with juvenile myoclonic epilepsy. Neurology.

[74] Ciumas C, Wahlin T-BR, Espino C, Savic I (2010). The dopamine system in idiopathic generalized epilepsies: identification of syndrome-related changes. Neuroimage.

[75] Odano I, Varrone A, Savic I, Ciumas C, Karlsson P, Jucaite A (2012). Quantitative PET analyses of regional [11C]PE2I binding to the dopamine transporter—application to juvenile myoclonic epilepsy. Neuroimage.

[76] Rocha L, Alonso-Vanegas M, Villeda-Hernández J, Mújica M, Cisneros-Franco JM, López-Gómez M (2012). Dopamine abnormalities in the neocortex of patients with temporal lobe epilepsy. Neurobiol Dis.

[77] Marsicano G, Goodenough S, Monory K, Hermann H, Eder M, Cannich A (2003). CB1 cannabinoid receptors and on-demand defense against excitotoxicity. Science.

[78] Wallace MJ, Blair RE, Falenski KW, Martin BR, DeLorenzo RJ (2003). The endogenous cannabinoid system regulates seizure frequency and duration in a model of temporal lobe epilepsy. J Pharmacol Exp Ther.

[79] Falenski KW, Blair RE, Sim-Selley LJ, Martin BR, DeLorenzo RJ (2007). Status epilepticus causes a long-lasting redistribution of hippocampal cannabinoid type 1 receptor expression and function in the rat pilocarpine model of acquired epilepsy. Neuroscience.

[80] Karlócai MR, Tóth K, Watanabe M, Ledent C, Juhász G, Freund TF (2011). Redistribution of CB1 cannabinoid receptors in the acute and chronic phases of pilocarpine-induced epilepsy. PLoS ONE.

[81] Chen K, Neu A, Howard AL, Földy C, Echegoyen J, Hilgenberg L (2007). Prevention of plasticity of endocannabinoid signaling inhibits persistent limbic hyperexcitability caused by developmental seizures. J Neurosci.

[82] Goffin K, Van Paesschen W, Van Laere K (2011). In vivo activation of endocannabinoid system in temporal lobe epilepsy with hippocampal sclerosis. Brain.

[83] Riaño Barros DA, McGinnity CJ, Rosso L, Heckemann RA, Howes OD, Brooks DJ (2014). Test-retest reproducibility of cannabinoid-receptor type 1 availability quantified with the PET ligand [^11^C]MePPEP. Neuroimage.

[84] Koepp MJ, Richardson MP, Brooks DJ, Duncan JS (1998). Focal cortical release of endogenous opioids during reading-induced seizures. Lancet.

[85] Bartenstein PA, Duncan JS, Prevett MC, Cunningham VJ, Fish DR, Jones AK (1993). Investigation of the opioid system in absence seizures with positron emission tomography. J Neurol Neurosurg Psychiatr.

[86] Hammers A, Asselin M-C, Hinz R, Kitchen I, Brooks DJ, Duncan JS (2007). Upregulation of opioid receptor binding following spontaneous epileptic seizures. Brain.

[87] McGinnity CJ, Shidahara M, Feldmann M, Keihaninejad S, Riaño Barros DA, Gousias IS (2013). Quantification of opioid receptor availability following spontaneous epileptic seizures: correction of [11C]diprenorphine PET data for the partial-volume effect. Neuroimage.

[88] De Fusco M, Becchetti A, Patrignani A, Annesi G, Gambardella A, Quattrone A (2000). The nicotinic receptor beta 2 subunit is mutant in nocturnal frontal lobe epilepsy. Nat Genet.

[89] Steinlein OK, Mulley JC, Propping P, Wallace RH, Phillips HA, Sutherland GR (1995). A missense mutation in the neuronal nicotinic acetylcholine receptor alpha 4 subunit is associated with autosomal dominant nocturnal frontal lobe epilepsy. Nat Genet.

[90] Picard F, Bruel D, Servent D, Saba W, Fruchart-Gaillard C, Schöllhorn-Peyronneau M-A (2006). Alteration of the in vivo nicotinic receptor density in ADNFLE patients: a PET study. Brain.

[91] Löscher W, Hirsch LJ, Schmidt D (2015). The enigma of the latent period in the development of symptomatic acquired epilepsy—traditional view versus new concepts. Epilepsy Behav.

